# RNA-Seq Analysis of Differential Gene Expression Responding to Different Rhizobium Strains in Soybean (*Glycine max*) Roots

**DOI:** 10.3389/fpls.2016.00721

**Published:** 2016-05-30

**Authors:** Songli Yuan, Rong Li, Shuilian Chen, Haifeng Chen, Chanjuan Zhang, Limiao Chen, Qingnan Hao, Zhihui Shan, Zhonglu Yang, Dezhen Qiu, Xiaojuan Zhang, Xinan Zhou

**Affiliations:** ^1^Key Laboratory of Oil Crop Biology, Ministry of AgricultureWuhan, China; ^2^Oil Crops Research Institute of Chinese Academy of Agriculture SciencesWuhan, China

**Keywords:** Soybean, symbiotic specificity, different nodulation phenotypes, RNA-seq, differential gene expression responding

## Abstract

The root nodule symbiosis (RNS) between legume plants and rhizobia is the most efficient and productive source of nitrogen fixation, and has critical importance in agriculture and mesology. Soybean (*Glycine max*), one of the most important legume crops in the world, establishes a nitrogen-fixing symbiosis with different types of rhizobia, and the efficiency of symbiotic nitrogen fixation in soybean greatly depends on the symbiotic host-specificity. Although, it has been reported that rhizobia use surface polysaccharides, secretion proteins of the type-three secretion systems and nod factors to modulate host range, the host control of nodulation specificity remains poorly understood. In this report, the soybean roots of two symbiotic systems (*Bradyrhizobium japonicum* strain 113-2-soybean and *Sinorhizobium fredii* USDA205-soybean)with notable different nodulation phenotypes and the control were studied at five different post-inoculation time points (0.5, 7–24 h, 5, 16, and 21 day) by RNA-seq (Quantification). The results of qPCR analysis of 11 randomly-selected genes agreed with transcriptional profile data for 136 out of 165 (82.42%) data points and quality assessment showed that the sequencing library is of quality and reliable. Three comparisons (control vs. 113-2, control vs. USDA205 and USDA205 vs. 113-2) were made and the differentially expressed genes (DEGs) between them were analyzed. The number of DEGs at 16 days post-inoculation (dpi) was the highest in the three comparisons, and most of the DEGs in USDA205 vs. 113-2 were found at 16 dpi and 21 dpi. 44 go function terms in USDA205 vs. 113-2 were analyzed to evaluate the potential functions of the DEGs, and 10 important KEGG pathway enrichment terms were analyzed in the three comparisons. Some important genes induced in response to different strains (113-2 and USDA205) were identified and analyzed, and these genes primarily encoded soybean resistance proteins, NF-related proteins, nodulins and immunity defense proteins, as well as proteins involving flavonoids/flavone/flavonol biosynthesis and plant-pathogen interaction. Besides, 189 candidate genes are largely expressed in roots and\or nodules. The DEGs uncovered in this study provides molecular candidates for better understanding the mechanisms of symbiotic host-specificity and explaining the different symbiotic effects between soybean roots inoculated with different strains (113-2 and USDA205).

## Introduction

The root nodule symbiosis (RNS) between legume plants and rhizobia is the most efficient and productive source of nitrogen fixation, and has critical importance in agriculture and mesology (Biswas and Gresshoff, 2014). Rhizobium-legume symbiosis is established under tight regulation to coordinate bacterial infection steps with re-activation of root cortical cells. The process is triggered by rhizobia-secreted specific lipo-chitin-oligosaccharide signal molecules to host secreted flavonoids, and accompanied by a series of signal transduction inside the root cells (Oldroyd and Downie, 2004, 2008; Libault et al., 2010; Hayashi et al., 2012b). Rhizobium-legume symbiosis is highly host-specific, meaning that one rhizobium strain could only establish a symbiotic system with a limited set of host plants and vice versa (Hayashi et al., 2012a). Such a symbiotic characteristic is very pronounced and has led to the definition of different legume–rhizobia associations, which always associated with distinct nodulation phenotype and/or symbiotic effects (Jones et al., 2008; Hayashi et al., 2012a).

The symbiotic specificity is determined by exchanging species- specific signals between a host plant and its symbiotic rhizobium (Perret et al., 2000). It is well known that rhizobia utilizes surface polysaccharides, secreted proteins/type III secretion system (T3SS) and nod factor to modulate host range (Lerouge et al., 1990; Schultze et al., 1992; Stacey, 1995; Bec-Ferte et al., 1996; Deakin and Broughton, 2009; Yang et al., 2010; Okazaki et al., 2013), while the mechanisms underlying the corresponding recognition of these rhizobial signals and compatibility control of the legume–rhizobia interaction in the host legume are not well understood. To unravel such mechanisms, it is critical to investigate the differences of nod factor signaling reception and transduction in the host legume inoculated with different rhizobia strains.

Soybean (*Glycine max*), one of the most important legume crops in the world, normally establishes a nitrogen-fixing symbiosis with different types of rhizobia, such as *Bradyrhizobium japonicum, Bradyrhizobium elkanii, Bradyrhizobium liaoningense, Bradyrhizobium yuanmingense, E. fredii/Sinorhizobium fredii, Rhizobium tropici, R. oryzae* and *Mesorhizobium tianshanense*, and the efficiency of symbiotic nitrogen fixation in soybean by application of inoculates greatly depends on the symbiotic host-specificity (Yang et al., 2010; Hayashi et al., 2012a). The various species of *Rhizobium* make up two broad groups of fast- and slow-growing strains based on their growth rate and other characteristics (Sadowsky and Bohlool, 1986). The best studied rhizobium-soybean symbiotic models are *B. japonicum*-soybean (Hennecke, 1990; Meakin et al., 2006; Wei et al., 2008; Mesa et al., 2009; Quelas et al., 2010; Tang et al., 2016) and *S. fredii*-soybean (Annapurna and Krishnan, 2003; Krishnan et al., 2011; Margaret et al., 2011; Jiao et al., 2016), while the differences of the molecular events of nodulation in soybean inoculated with these two rhizobia strains remain unclear.

In most cases, soybean genotypes restrict nodulation with their specific strains (or sero-groups; Keyser and Cregan, 1987; Cregan et al., 1989) and several dominant genes (Rj2, Rj3, Rj4, and Rfg1) have been designated to control the host specificity in the soybean–rhizobia symbiosis (Kanazin et al., 1996; Yang et al., 2010). Besides, comparative analysis of genome sequences of six legumes revealed a large number of symbiotic genes in soybean (Zhu et al., 2013) and RNA-Seq transcription data predicted several nodulation-related gene regulatory networks (Zhu et al., 2013). However, these results cannot explain why different symbiotic effects existed among different soybean–rhizobia associations. To improve our understanding of the host legume control of nodulation specificity, we (1) investigated the molecular events of nodulation in soybean roots inoculated with *B. japonicum* strain 113-2 or *S. fredii* strain USDA205, (2) identified a large number of differentially expressed genes (DEGs), and (3) analyzed the DEGs that are associated with the flavonoids biosynthesis pathway and the plant-pathogen interaction pathway. Our results provide fundamental clues to the mechanisms underlying the host-specific manners of rhizobial signals reception and transduction and shed new light on the host legume control of nodulation specificity.

## Materials and methods

### Plant materials and growth conditions

Seeds of Soybean Tian long No.1 (stored in our lab) were surface-sterilized and germinated on moistened filter paper for 2–3 d at 28°C in an incubator with 70% relative humidity (RH) and a 16-h light/8-h dark photoperiod. They were then grown in pots filled with sterilized vermiculite and perlite (1:1) supplemented with half-strength B&D medium in a chamber with a 16/8 h day/night cycle at 28°C for 1–2 day before inoculation with rhizobium strain113-2 (stored in our lab) and USDA205 (provided by Huazhong Agricultural University in China). After inoculation, plants were kept under the same growth conditions. Their growth situations at 12, 30, and 42 days of post-inoculation (dpi) and roots at 12 and 42 dpi were photographed. Chlorophyll contents in the first trifoliolate leaf were measured by SPAD-502 Plus Chlorophyll Meter. The mean values of nodule number and nodule dry weight were calculated using software SPSS Statistics 17.0.

Samples for RNA isolation were collected from soybean roots (1) at 0.5 h; (2) 7 h/24 h; (3) 5 day; (4) 16 day and (5) 21 day of post inoculation. The former three time points represent the period that soybean root hairs recognize the rhizobium signals, the period that soybean root hairs are infected by Rhizobium (root hairs curling at 7 h of post inoculation (hpi) and cortical cells dividing at 24 hpi) and the nodule primordia formation period, respectively, and the latter two time points represent two early nodule development periods. Samples collected at 7 and 24 hpi were mixed as one sample. Each collection was performed with three biological replicates. RNAs isolated from the three replicates were mixed at 1:1:1 ratio for subsequent library construction and sequencing.

### RNA extraction and cDNA library preparation

Total RNA was isolated using TRIzol reagent (Invitrogen, USA) and stored in a -80°C for downstream gene-expression analysis. Potential genomic DNA were removed using RNeasy plant mini kit (QIAGEN, Germany) and RNA quantity and quality were measured using an Epoch Multi-Volume Spectrophotometer system, NanoDrop and Agilent 2100 Bioanalyzer (Agilent Technologies, Palo Alto, CA, USA). All samples had A_260_/A_280_ and A_260_/A_230_ ratios of 2.13~2.23, RIN value above 8.5 and 28S/18S above 1.6 except one.

To obtain a comprehensive range of transcripts, an equal amount of total RNA from each sample was pooled for RNA-Seq. mRNAs were enriched using oligo (dT) magnetic beads, fragmented in fragmentation buffer to about 200 bp and reverse-transcribed into single strand cDNA using random hexamer primers. After RNaseH digestion, the cDNA were converted into double strand cDNAs with DNA polymerase I and purified using magnetic beads. After end repair and addition of single nucleotide A (adenine) at 3′-end, cDNA were ligated to adaptors and prepared as libraries. After qualification and quantification using an Agilent 2100 Bioanaylzer and ABI Step One Plus Real-Time PCR System, the libraries were subjected to sequencing on Illumina HiSeq™ 2000.

### Clean reads library formation

Raw reads which include partial adaptor sequences and/or low quality reads were generated from the original image data from Illumina Hi Seq™ 2000 and filtered into high quality (clean) reads after (1) trimming off the adaptor sequences; (2) eliminating the reads with higher than 10% unknown bases and reads with higher than 50% low quality bases (base with quality value ≤ 5). The clean reads were then mapped to reference genes and genome (ftp://ftp.jgi-psf.org/pub/compgen/phytozome/v9.0/Gmax/annotation/Gmax_189_transcript.fa.gz and ftp://ftp.jgi-psf.org/pub/compgen/phytozome/v9.0/Gmax/assembly/Gmax_189.fa.gz) using SOAP aligner/SOAP2 (Li et al., 2009) with threshold that no more than two mismatches were permitted in the alignment. The mapping results are shown in Supplemental Table S2.

### Function annotation and pathway analysis

To understand function distribution of genes at macro-level, gene ontology (GO) annotation of DEGs was performed using software Blast2GO and used for GO functional classification of DEGs using software WEGO.

Kyoto Encyclopedia of Genes and Genomes (KEGG) is the major public pathway-related database, the significant differences between different groups were calculated using the formula

$$P=1-\sum_{i=0}^{\text{m}=1}\frac{\left(\frac{M}{i}\right)\left(\frac{N-M}{n-i}\right)}{\left(\frac{N}{n}\right)}$$

Where N is the number of all genes with KEGG annotation, n is the number of DEGs in N, M is the number of all genes annotated to specific pathways, and m is the number of DEGs in M. Besides the list of the most meaningful pathways, the detailed pathway information in KEGG database can be obtained by clicking the hyperlinks on KEGG pathways. The gene identifiers mapped to the pathway were used as the gene sets, and the lists of DEGs in each of the comparisons were used as the test sets.

### Quantitative real-time PCR (qPCR)

DEGs were further evaluated using qPCR. In brief, RNA samples were treated with DNase I (Takara) and reverse-transcribed using a Prime Script RT reagent Kit (Perfect Real Time) with gDNA Eraser (Takara Bio, Inc) and oligo (dT) as the primer. cDNA from the reverse transcription of approximately 1 μg of RNA was used as the template for qPCR using primer sets listed in Supplemental Table S5 and cycling conditions of 30 s at 95°C followed by 40 cycles of 5 s at 95°C, 15 s at 60°C and 12 s at 72°C and final 5 s at 72°C. The polyubiquitin transcript was used as the internal control. Sample cycle threshold (CT) values were standardized for each template using the reference gene as control, and the 2^−Δ*ΔCT*^ method was used to analyze the relative changes in gene expression from the qPCR experiments. Three replicate reactions per sample were used to ensure statistical credibility.

## Results

### Symbiotic phenotypic characterization of soybean inoculated with rhizobium strains113-2 or USDA205 at roots

**Figure 1:**
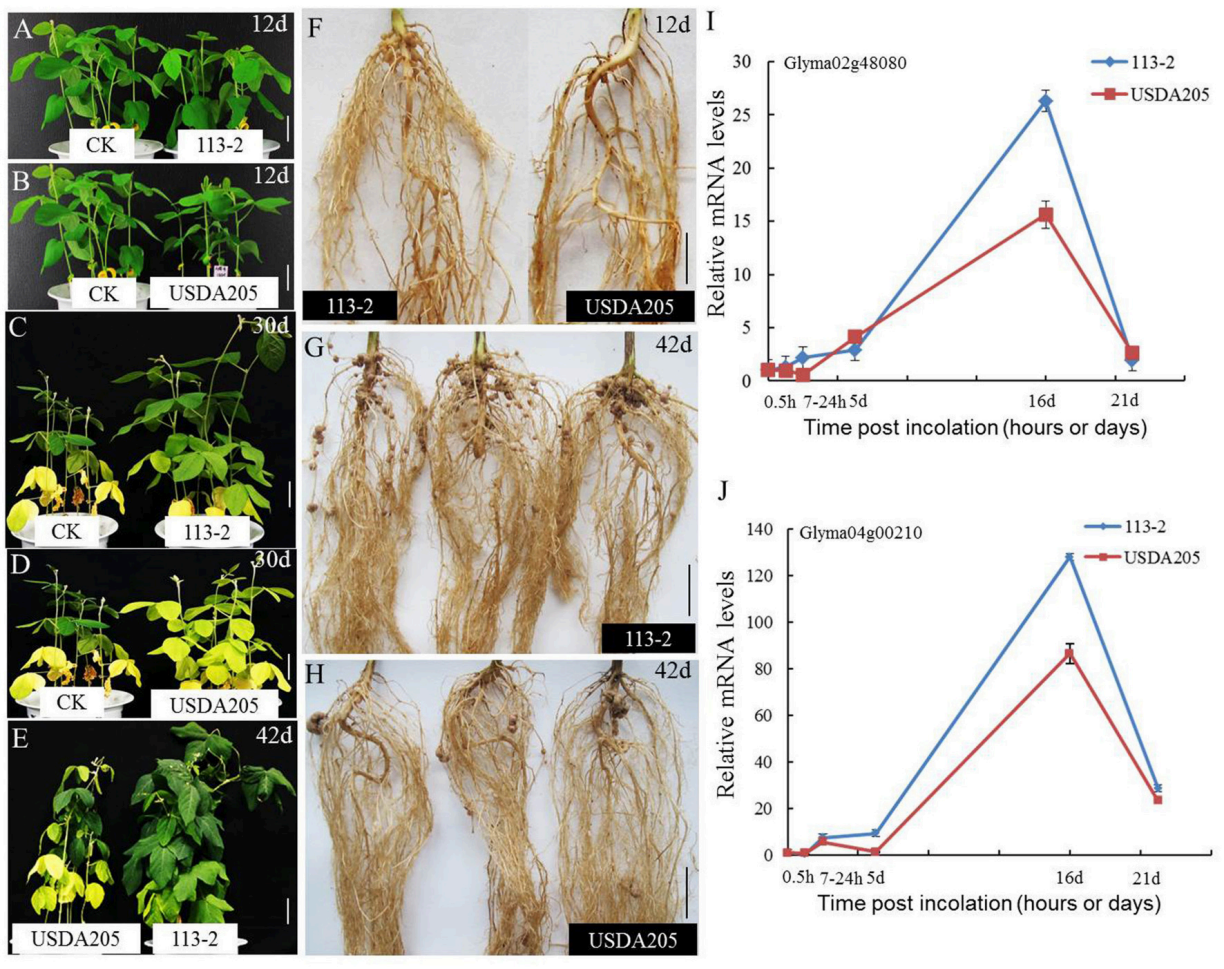
**Symbiotic phenotypic features of *B. japonicum* 113-2-soybean and *S. fredii* USDA205-soybean symbionts**. Slow-growing rhizobium strains *B. japonicum* 113-2 was originated from southern China and fast- growing rhizobium strains *S. fredii* USDA205 was from USA. The soybean cultivar is Tian long No.1 (China). **(A–E)** The growth of plants without inoculation (12 and 30 day) and the two symbiosis (12, 30, and 42 day). **(F–H)** Nodulation phenotypes were examined at 12 and 42 day after inoculation with 113-2 or USDA205. **(I,J)** The expression levels of *GmNIN*-like genes (Glyma02g48080 and Glyma04g00210) in soybean roots at five time points (0.5 h, 7–24 h, 5 day, 16 day, and 21 day) after inoculation with rhizobium strains113-2 or USDA205. Bars, 4 cm **(A,B,D)**; 4.5 cm **(C)**; 5.0 cm **(E,F,G,H)**; d, days; h, hours.

Soybean can establish nitrogen-fixing symbiosis with different species of rhizobium strains (Hayashi et al., 2012a). The best studied rhizobium-soybean symbiotic models are *B. japonicum*—soybean and *S. fredii*—soybean (Israel et al., 1986; Annapurna and Krishnan, 2003; Sanz-Saez et al., 2015). The symbiotic phenotype was different between soybean Tian long No.1 inoculated with slow-growing rhizobium strains *B. japonicum* 113-2 and fast-growing rhizobium strains *S. fredii* USDA205 (Figure 1, Supplemental Table S1). At 12 dpi, the control (CK, inoculated with media only) had slightly better growth and higher chlorophyll content than the two symbionts (Figures 1A,B, Supplemental Table S1), mainly because the symbiotic process needed more energy from the host plant and the nitrogen fixation function was weak during this period. At 30 and 42 dpi (Figures 1C,D, Supplemental Table S1), the control had no additional nitrogen supply, thus its growth and chlorophyll synthesis was limited by insufficient nitrogen in cotyledon. Comparison of the two symbionts showed that *S. fredii* USDA205-soybean symbiont had much less nodule numbers per root system (0 vs. 13) at 12 dpi (Figure 1F, Supplemental Table S1), although they had similar growth and chlorophyll content (Figures 1A,B, Supplemental Table S1). In addition, compared with *B. japonicum* 113-2-soybean symbiont, *S. fredii* USDA205-soybean symbiont had (1) obviously more yellow leaves and less chlorophyll content at 30 dpi (Figures 1C,D, Supplemental Table S1); (2) worse growth (Figure 1E), less chlorophyll content (Supplemental Table S1), fewer nodule per root system (16.64 vs. 51.52, Figure 1G) and larger nodules (3-fold) (Figure 1H, Supplemental Table S1) at 42 dpi, and (3) lower expression of *Gm NIN-like* genes (Glyma02g48080 and Glyma04g00210), which are required for nodulation (Borisov et al., 2003), in soybean roots at almost all the tested time points (Figures 1I,J). These results suggest that the soybean roots of these two symbiotic systems have differential cellular responses.

### RNA-seq quality assessment and identification of DEGs

The above-mentioned different symbiotic phenotypes are related to the symbiotic host-specificity and maybe mainly due to the molecular events of nodulation (Marioni et al., 2008). To investigate the causes of these different symbiotic phenotypes, RNA-Seq was performed for soybean root samples at five important time points: 0.5, 7–24 hpi, 5, 16, and 21 dpi. The statistic results of alignment (Supplemental Table S2), randomness assessment (Supplemental Figure S1), the proportion of clean reads among the total acquired reads was more than 99.6%, and sequencing saturation analysis (Supplemental Figure S2) indicated that the sequencing was of good quality and contained sufficient information for gene expression analysis.

To judge the significance of differences of DEGs in soybean roots inoculated with different rhizobium strains, the false discovery rate (FDR) ≤ 0.001 and |log2 ratio| ≤ 1 were used as criteria for the following three comparisons: (1) comparison between soybean roots uninoculated vs. inoculated with rhizobium strains113-2 at 0.5, 7–24 hpi, 5, 16, and 21 dpi (Group 1); (2) comparison between soybean roots uninoculated vs. inoculated with rhizobium strain USDA205 at 0.5, 7–24 h, 5, 16, and 21 day post inoculation (Group 2); and (3) comparison between soybean roots inoculated with rhizobium strain USDA205 vs. inoculated with rhizobium strain 113-2 at 0.5, 7–24 h, 5, 16, and 21 day post inoculation (Group 3). DEGs in these comparisons are shown in Supplemental Table S3.

**Figure 2:**
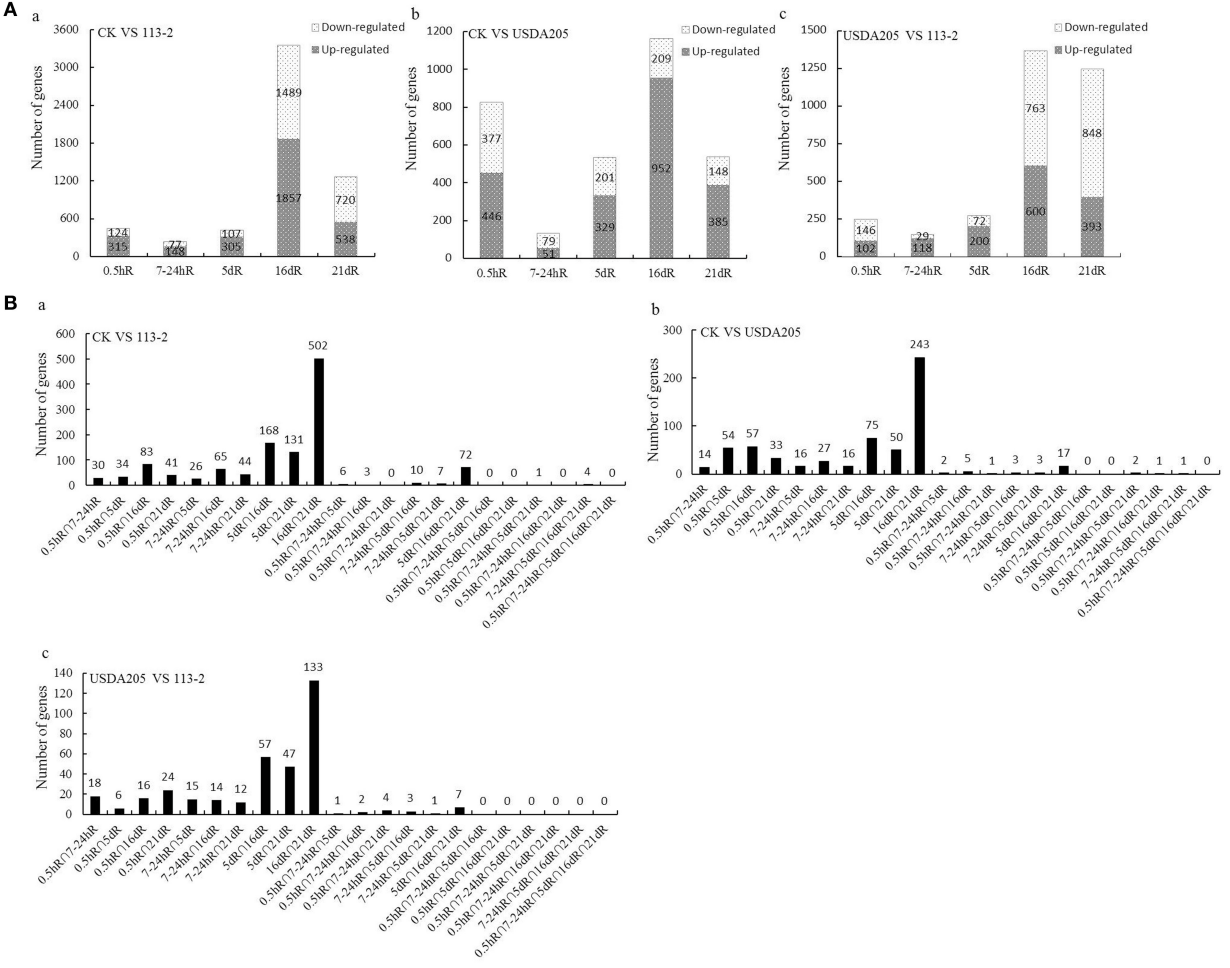
**Genes differentially expressed in soybean roots at five time points in the three Groups (CK vs. 113-2, CK vs. USDA205, and USDA205 vs. 113-2). (A)** Genes differentially expressed in soybean roots at different time points were separated into two groups according to whether they were significantly up-regulated or down-regulated. a, CK vs. 113-2 (Group 1); b, CK vs. USDA205 (Group 2); c, USDA205 vs. 113-2 (Group 3). **(B)** The numbers of differentially expressed genes in 22 gene sets in the three groups. Five different post-inoculation time points (0.5 h, 7–24 h, 5 d, 16 d, and 21 d) are included and the division of DEGs into different gene sets depends on which time points (two or more) the DEGs were identified. a, CK vs. 113-2 (Group 1); b, CK vs. USDA205 (Group 2); c, USDA205 vs. 113-2 (Group 3).

The numbers of up-regulated and down-regulated DEGs in the three comparisons are shown in Figure 2A. The number of DEG at 16 dpi was the highest in Group 1 (that was 3346) and Group 2 (that was 1161), indicating the beginning of a series of new processes. Most of the DEGs in Group 3 were found at 16 dpi and 21 dpi, indicating that most of the differential gene expression responses in soybean roots to rhizobia strains 113-2 and USDA205 happened at 16 and 21 dpi. The numbers of DEGs found at two or more time points in the three comparisons were showed in Figure 2B. Twenty two gene sets were analyzed and DEGs in 16dR∩21dR gene set was proportional to the highest number, besides, there were no DEGs consistently found at five time points (0.5hR∩7-24hR∩5dR∩ 16dR∩21dR) in the three comparisons.

### Function ontology and KEGG pathway enrichment analysis of DEGs

**Figure 3:**
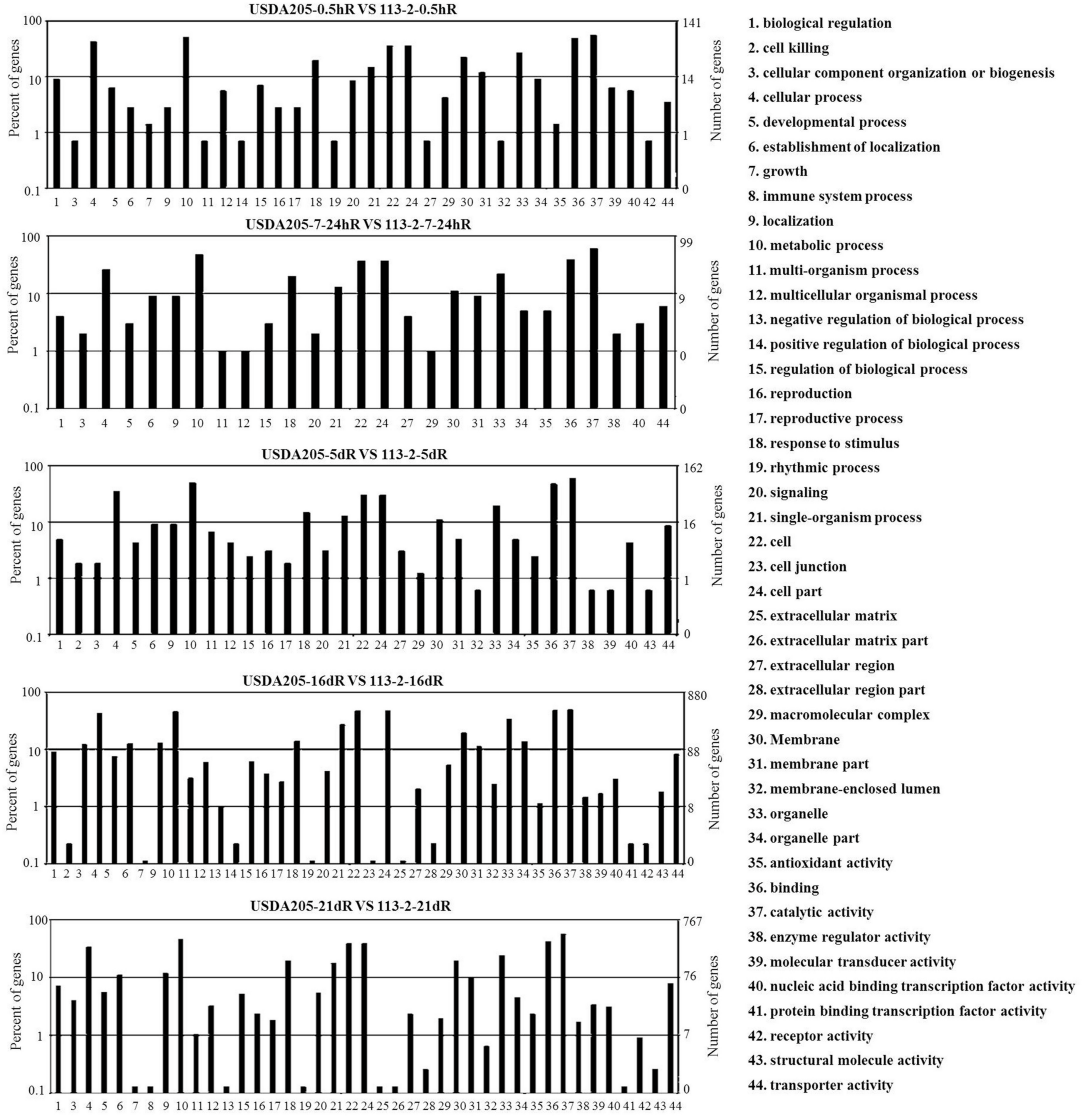
**Gene ontology-based functional annotation of DEGs in soybean roots at five post inoculation time points in USDA205 vs. 113-2 (Group 3)**. The gene category frequencies and the number of genes in each term were shown in histograms. 44 go function terms were indicated and divided into three categories—biological process (1–21), cellular components (22–34), and molecular function (35–44), and 26 go function terms(1, 3–6, 9–12, 15, 18, 20–22, 24, 27, 29–31, 33–37, 40, 44) were indicated at all the five tested time points.

To evaluate the potential functions of the DEGs between the two symbiotic systems, DEGs with > 2-fold expression change in Group 3 were assigned to different GO categories such as biological process, molecular function, and cellular location, and 44 functional GO terms were analyzed (Figure 3). For all the five tested time points, the biological processes associated with the DEGs mainly focused on metabolic process, cellular process, response to stimulus and single-organism process. The cellular components mainly included cell, cell part and organelle. The main molecular functions of the DEGs were binding and catalytic activity. Most of the DEGs involved in these 44 functional GO terms were identified in USDA205-16dR vs. 113-2-16dR and USDA205-21dR vs. 113-2-21dR, moreover, only two GO terms (immune system process and extracellular matrix part) were not found in USDA205-16dR vs. 113-2-16dR and three (cell killing, positive regulation of biological process and cell junction) were not in USDA205-21dR vs. 113-2-21dR. Besides, 26 go function terms were indicated at all the five tested time points and revealed no high shift in the distribution (Figure 3).

**Figure 4:**
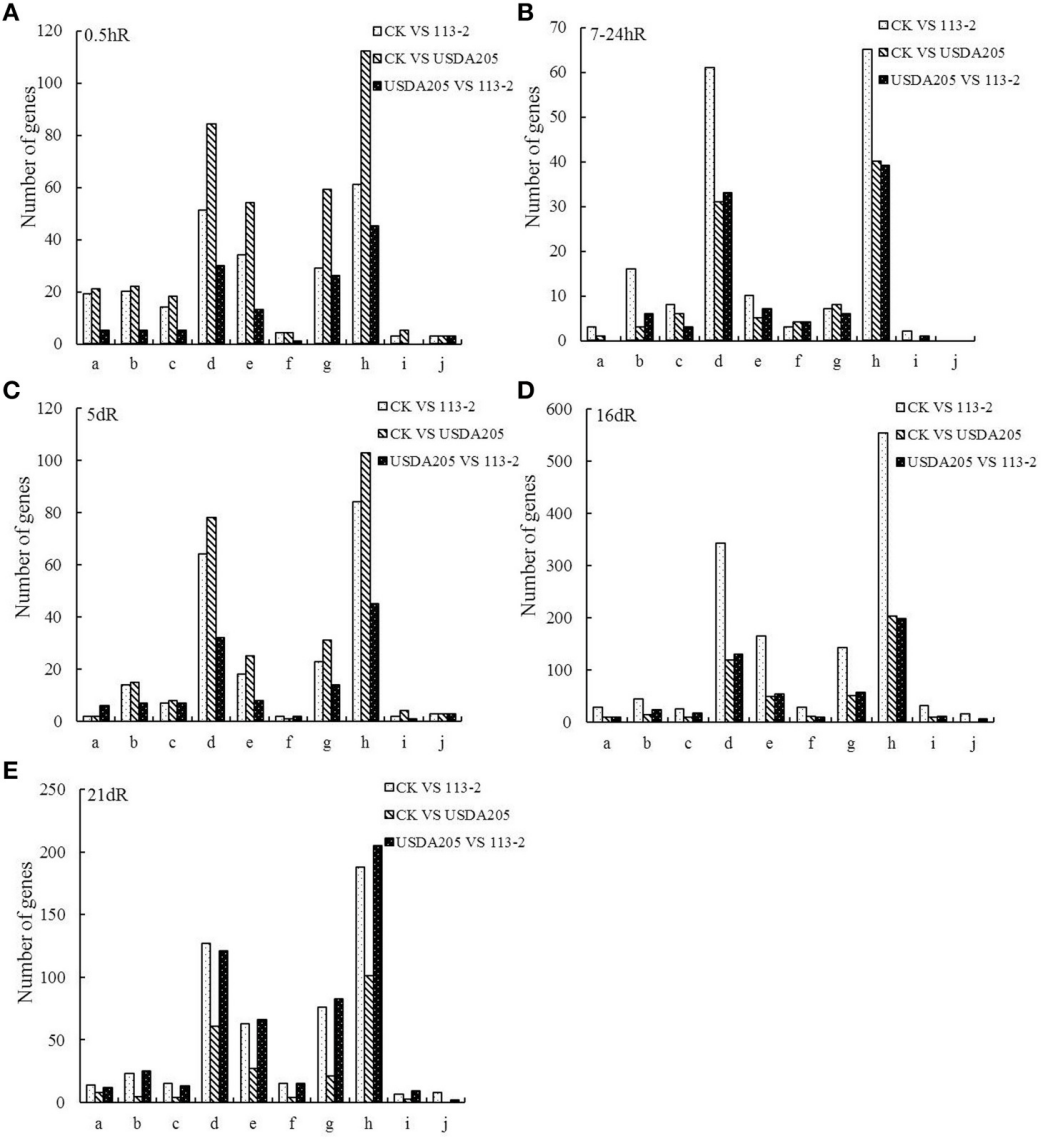
**KEGG pathway enrichment analyses of DEGs for 10 KEGG pathways in the three groups**. The x- and y-axes represent pathway categories and the number of genes in each pathway, respectively. a, ABC transporters; b, Flavonoid biosynthesis; c, Flavone and flavonol biosynthesis; d, Biosynthesis of secondary metabolites; e, Plant hormone signal transduction; f, Nitrogen metabolism; g, Plant-pathogen interaction; h, Metabolic pathways; i, Ubiquitin mediated proteolysis; j, RNA transport.

KEGG is the major public pathway-related database. Therefore, we analyzed 10 KEGG pathways (Figure 4). In these pathways, the metabolic pathways were most prominent, followed by biosynthesis of secondary metabolites. Other two pathways: plant hormone signal transduction and plant-pathogen interaction, were also main enrichment pathways in the three groups. More DEGs at 7–24 hpi, 16 and 21 dpi in Group1 were involved in these 10 KEGG pathways than those in Group 2 (Figures 4B,D,E). By contrast, more DEGs at 0.5 and 5 dpi in Group 2 were involved in these 10 KEGG pathways than those in Group 1 (Figures 4A,C). Moreover, most of DEGs in Group 3 involved in these 10 KEGG pathways were identified at 16 and 21 dpi (Figures 4D,E).

### DEGs associated with the flavonoids/flavone/flavonol biosynthesis pathway and the plant-pathogen interaction pathway

In order to evaluate whether the recognition of different rhizobia to host legume is related to the secreted flavonoids, we analyzed the DEGs associated with the flavonoids biosynthesis pathway and flavone and flavonol biosynthesis pathway in Group 1 and Group 2 at 0.5 hpi in more detail (Table 1). The results showed that changes in expression levels of 28 DEGs were significantly different between the two groups (Table 1), more DEGs and higher change folds were found in Group 2 than Group 1, indicating that the flavonoids/flavone/flavonol biosynthesis pathways are more sensitive to the surface substance produced by USDA205.

**Table 1:** **The DEGs associated with flavonoids biosynthesis pathway in soybean at 0.5h of post inoculation based on log2 ratio**.

**Gene code**	**Group 1**	**Group 2**
Glyma01g29930		2.58
Glyma01g42350	1.6	
Glyma02g05470		−3.48
Glyma02g42180	1.8	
Glyma02g42470		1.8
Glyma03g07680	2.6	3.4
Glyma04g04270	1	
Glyma05g36210		1.48
Glyma06g43970		2
Glyma07g18280		4
Glyma08g19290		−1.2
Glyma08g42440	−2.2	−3.4
Glyma08g42450		−1.9
Glyma10g16790		−1.2
Glyma11g05680		−1.5
Glyma13g37810		−1.4
Glyma14g04800		−1.1
Glyma14g06710	1.2	
Glyma15g38670		−1.4
Glyma16g03760		−1.4
Glyma18g12180		−1.4
Glyma18g12210		−1.1
Glyma18g12280		−2.2
Glyma18g40186	−2.2	
Glyma18g49240	−1.3	−2.1
Glyma19g03760	−2.5	−4.3
Glyma19g03770	−2.3	−3.5
Glyma19g37116		−1

**Figure 5:**
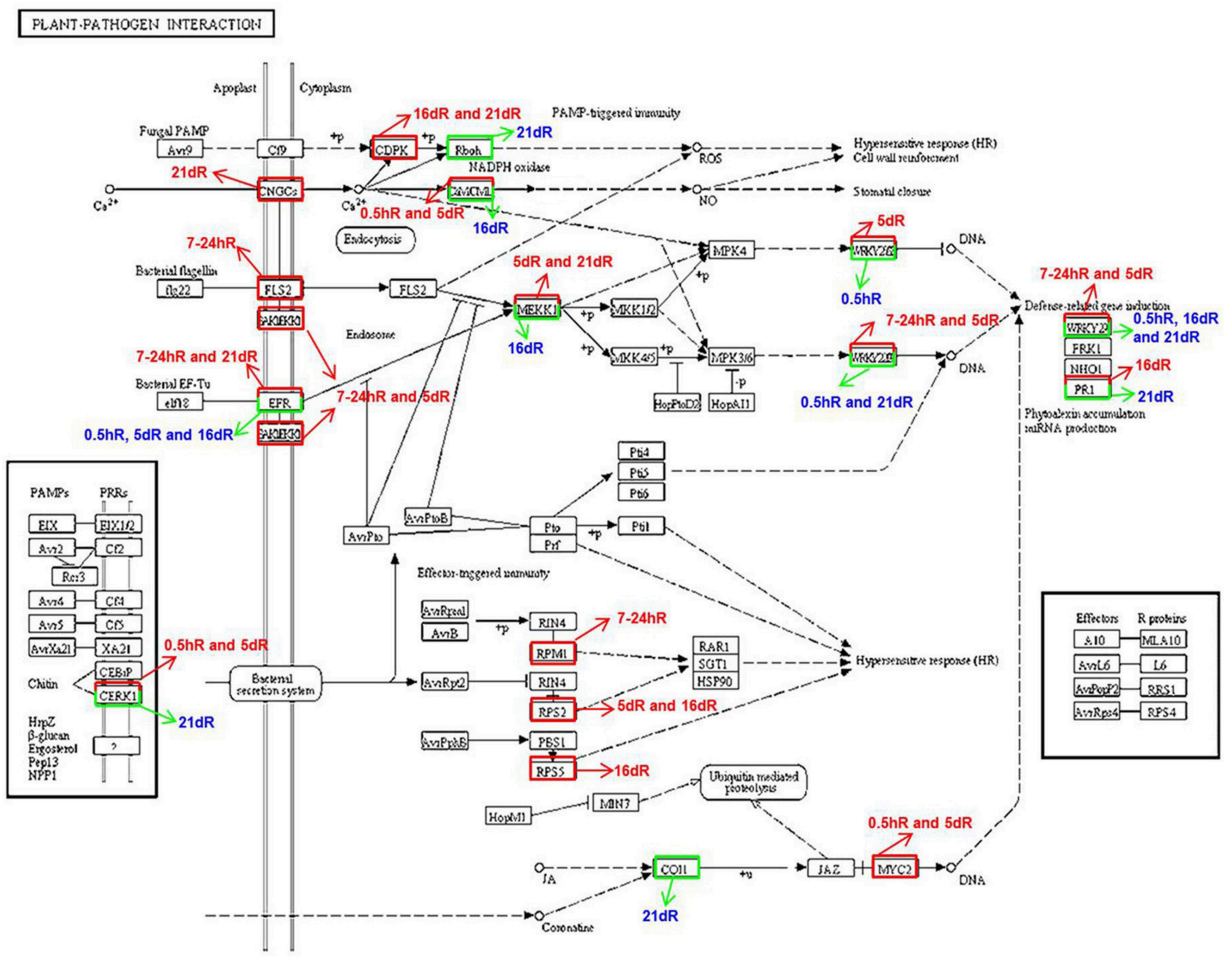
**Differentially expressed genes associated with the plant–pathogen interaction pathways in soybean roots at five time points after inoculation with USDA205 or 113-2**. DEGs in Group 3 (USDA205 vs. 113-2, comparison between soybean roots inoculated with rhizobium strain USDA205 vs. inoculated with rhizobium strain 113-2 at 0.5 h, 7–24 h, 5 d, 16 d, and 21 d post inoculation), associated to the KEGG plant–pathogen interaction pathway in the KEGG database. Up-regulated genes are boxed in red, down-regulated genes are boxed in green. The red arrows point out the up-regulation of DEGs, the green arrows point out the down-regulation of DEGs.

In the absence of Nod factor signal, legume plants terminate the infection process perhaps via a defense response (Jones et al., 2008). To explore the differential cell defense responses between soybean roots inoculated with rhizobium strains 113-2 and USDA205, the plant–pathogen interaction KEGG pathway was analyzed in more detail in Group 3 (Figure 5, Table 2). Among the DEGs annotated to this pathway, 7 were identified at 0.5 hpi, of which 4 were down-regulated; 6 were identified at 7–24 hpi, all of which were up-regulated; 10 were identified at 5 dpi, of which only 1 was down-regulated; 8 were identified at 16 dpi, of which 4 were down-regulated, and 10 were identified at 21 dpi, of which 6 were down-regulated (Figure 5), and the detailed expression information of 55 important DEGs associated with this pathway in Group 3 was shown in Table 2. These results indicated differential defense responses in soybean roots is related to the process of soybean responding to rhizobium strains 113-2 and USDA205.

**Table 2:** **The DEGs associated with the plant–pathogen interaction pathway in group3 (USDA205 vs. 113-2) based on the log2 ratio**.

**Gene**	**Gene code in soybean**	**0.5hR**	**7-24hR**	**5dR**	**16dR**	**21dR**
*CERK1*	Glyma11g06740	1.1				
	Glyma13g39880			1.3		
	Glyma17g36630					−1.6
*MYC2*	Glyma06g09670	2				
	Glyma08g21130	1				
	Glyma12g33751			1.4		
*CALM*	Glyma19g41730	1.4				
	Glyma05g07720			1.7	−1.4	
*EFR*	Glyma09g35011	−6.2				
	Glyma05g25360	−1.1				
	Glyma08g08360		1.5	−1.5		
	Glyma04g40080				−1	
	Glyma09g35090					1.2
	Glyma06g25110					1.2
*WRKY25*	Glyma05g25770	−1.7				
	Glyma03g05220	−1.6				
	Glyma01g31921	−1				
	Glyma15g00570	−1				
	Glyma18g16170			3.3		
*WRKY29/22*	Glyma05g37390	−1.4				
	Glyma05g36970	−1.3				
	Glyma09g06980	−1.3				
	Glyma08g02580	−1.3				
	Glyma13g00380	−1.2				
	Glyma17g06450	−1.2				
	Glyma04g41701	−1				
	Glyma19g44380		1.4			−1.3
	Glyma16g02960		1.1			
	Glyma09g24080				−1.4	
	Glyma03g00460			1.9	−1.3	
	Glyma03g41750					−1.6
*FLS2*	Glyma16g27260		1.8			
*BAK1*	Glyma17g07440		3.7			
	Glyma16g32600			4.2		
	Glyma09g27600			4		
*RPM1*	Glyma09g34381		1			
*MEKK1P*	Glyma14g08801			1		1
	Glyma19g42340				−1.1	
	Glyma01g39380					2.1
*CDPK*	Glyma10g30940				1.2	
	Glyma11g34000					1.1
*PR1*	Glyma15g06830				3.2	
	Glyma13g32510				2.9	
	Glyma15g06780				1.1	−1.9
	Glyma13g32560					−3.4
	Glyma15g06770					−2.9
	Glyma13g01250					−2.8
	Glyma15g06790					−2.3
*RPS2*	Glyma01g31550				1.2	
	Glyma05g17470			1.2	1.2	
*RPS5*	Glyma01g05710				5.9	
*CNGF*	Glyma17g12740					1.1
	Glyma08g24960					1.1
*RBOH*	Glyma05g00420					−1.2
*COI-1*	Glyma18g03420					−1

### Analysis of DEGs encoding resistance proteins in soybean roots inoculated with rhizobium strains 113-2 or USDA205

**Figure 6:**
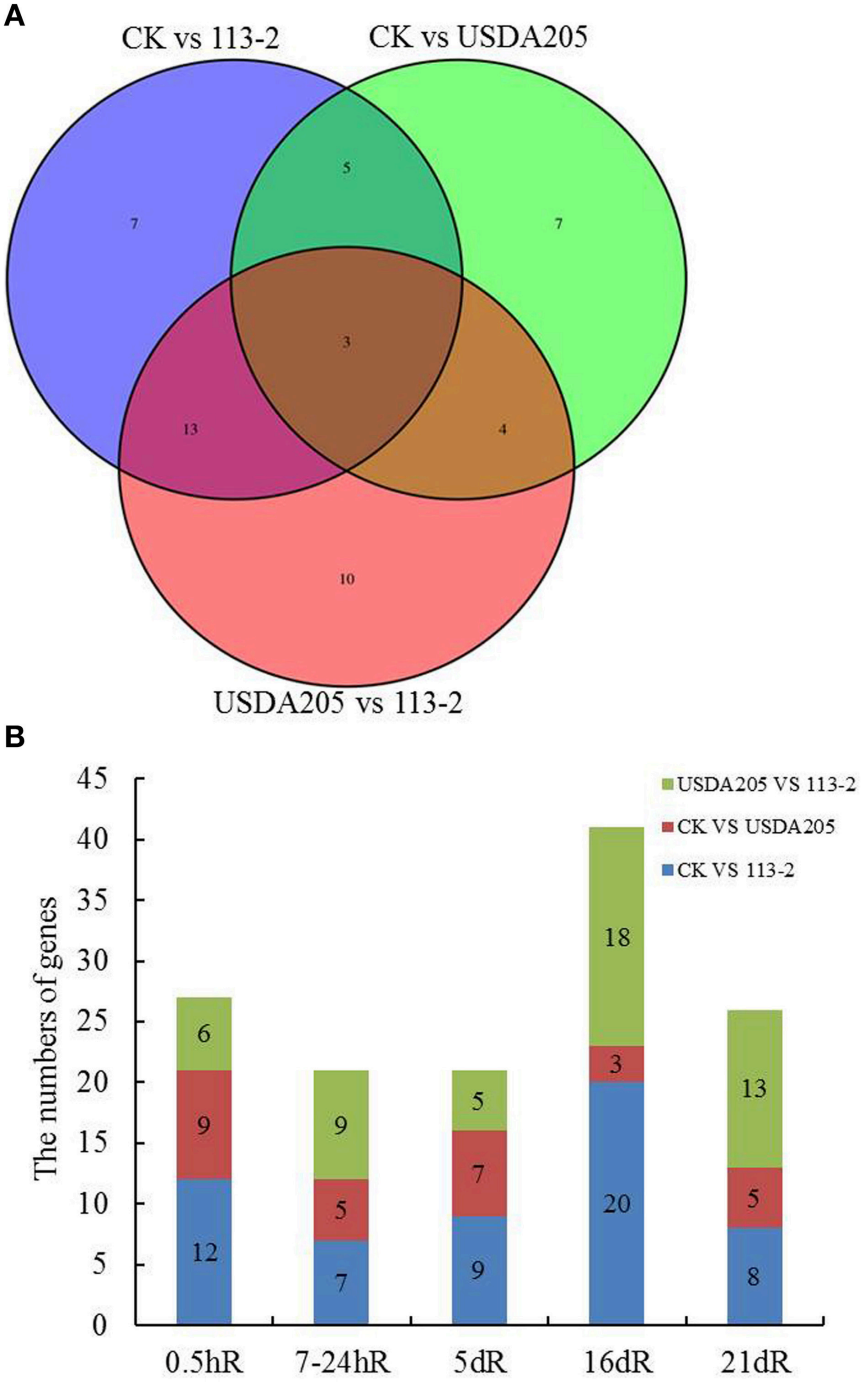
**DEGs encoding resistance (R) proteins in soybean roots inoculated with rhizobium strains 113-2 or USDA205. (A)** Venn diagram showing the numbers of DEGs encoding resistance (R) genes in soybean roots in the three groups (CK vs. 113-2, CK vs. USDA205 and USDA205 vs. 113-2). **(B)** Numbers of R genes in soybean roots at five different post inoculation time points in the three groups.

The genetic loci of soybean, namely Rj (s) or rj (s), have been identified responsive to the nodule formation (Hayashi et al., 2012a), and several of these genes (e.g., Rj2, Rj4, and Rfg1) are found involving in restrict nodulation with specific rhizobial strains (Yang et al., 2010). To investigate whether resistance (R) proteins are involved in control of the nodulation phenotypes in soybean inoculated with rhizobium strains 113-2 or USDA205, DEGs encoding R proteins in soybean roots were analyzed in more detail (Figure 6). Figure 6A shows the numbers of DEGs encoding R proteins in each set. A total of 24 such genes were only found in one group (7, 7, and 10 in Groups 1–3, respectively), 22 genes were found in two groups (5 in Group 1 and 2; 4 in Group 2 and 3; 13 in Group 1 and 3) and 3 genes (Glyma05g17470, Glyma12g01420, and Glyma19g35270) were found in all three groups. Figure 6B shows the number of R genes differentially expressed at the five different time points in soybean roots in the three groups. It can be seen that more R genes were found in Group 1 than in Group 2 at each time point, especially at 16 dpi and not all differentially expressed R genes in Group 1 and/or Group 2 are also found in Group 3 (Figures 6A,B), indicating that they participate in nodulation but not in restriction of specific rhizobial strains. Besides, some differentially expressed R genes in Group 3 were not found in Group 1 and Group 2, indicating that these R genes may be associated with T3SS of rhizobium and/or defense responses in soybean roots.

### Analysis of selected nodulation factor (NF)-related genes and nodulin genes in soybean roots inoculated with different rhizobium strains 113-2 and/or USDA205

Twenty-five DEGs responsible for broad NF signal pathway and nodulation were identified in three groups by searching for homologs of *M. truncatula* and *L. japonicus* NF-related genes in soybean genome sequence database (Table 3; Kevei et al., 2007; Mbengue et al., 2010; Schmutz et al., 2010; Kim et al., 2013). Because same proteins may be encoded by one or more DEGs, only 14 NF-related genes are listed (Table 3), and the identified orthologs in these three legumes were named according to the given nomenclature in cloning studies in *M. truncatula, L. japonicus* and soybean.

**Table 3:** **List of 14 NF-related genes in soybean roots inoculated with rhizobium strains 113-2 or USDA205**.

**Gene name**	***M. truncatula***	***L. japonicus***	***Glycine max***	**Group 3**	**Group 2**	**Group 1**
*MtERN1*	Medtr7g102550	Lj1.CM0104.2670.r2.m	Glyma16g04410	Glyma16g04410		
	Medtr6g031080		Glyma19g29000		Glyma19g29000	
*MtFLOT2*	Medtr3g137870		Glyma06g06930	Glyma06g06930	Glyma06g06930	Glyma06g06930
	Medtr1g099720					
*MtIPD3*	Medtr5g027010	Lj2.CM0803.150.r2.m	Glyma01g35255			Glyma01g35255
			Glyma09g34695			Glyma09g34695
*MtLIN*	Medtr1g112060	Lj5.CM0909.400.r2.m	Glyma10g33851			Glyma10g33851
*MtLYR3*	Medtr5g019000	Lj2.CM0323.420.r2.d	Glyma02g06700		Glyma02g06700	
*MtNFP*	Medtr5g018990	Lj2.CM0323.400.r2.d	Glyma11g06740	Glyma11g06740	Glyma11g06740	
	Medtr8g093910					
*MtNIN*	Medtr5g106690	Lj2.CM0102.250.r2.m	Glyma06g00240			Glyma06g00240
			Glyma04g00210	Glyma04g00210	Glyma04g00210	Glyma04g00210
			Glyma02g48080	Glyma02g48080	Glyma02g48080	Glyma02g48080
*MtNSP1*	Medtr8g025000	Lj3.CM0416.1260.r2.d	Glyma16g01020		Glyma16g01020	Glyma16g01020
	Medtr5g015580		Glyma05g22460	Glyma05g22460		Glyma05g22460
	Medtr8g101580					
*MtNSP2*	Medtr3g097800	Lj1.CM1976.90.r2.m	Glyma04g43090			Glyma04g43090
	Medtr5g065380		Glyma13g02840	Glyma13g02840		Glyma13g02840
*MtNup133*	Medtr5g097260	Lj2.CM0191.150.nc	Glyma14g01130			
			Glyma02g47560	Glyma02g47560		Glyma02g47560
*MtPUB1*	Medtr5g083030		Glyma02g43190	Glyma02g43190		
*MtHMGR1*	Medtr5g026500		Glyma11g09330		Glyma11g09330	Glyma11g09330
*GmN56*	Medtr1g146810	Lj5.CM0492.390.r2.m	Glyma19g29880		Glyma19g29880	Glyma19g29880
		Lj1.CM0001.650.r2.m	Glyma20g38950		Glyma13g12484	Glyma20g38950
		Lj1.CM0001.690.r2.m	Glyma13g12484			Glyma13g12484
		Lj1.CM0001.710.r2.m				
*GmENOD93*	Medtr8g119590		Glyma05g08400	Glyma05g08400	Glyma05g08400	Glyma05g08400
			Glyma06g24760	Glyma17g12610	Glyma17g12610	Glyma17g12610
			Glyma17g12610		Glyma06g24760	Glyma06g24760

Nodulins are legume genes whose expression is induced by rhizobium bacteria upon nodulation (Denance et al., 2014). Twenty-nine nodulin genes have been analyzed in *M. truncatula* and most of them play key roles in nodulation (Gamas et al., 1996; Denance et al., 2014). In this study, 25 soybean nodulin genes were identified as DEGs in soybean roots (Table 4). Among them, only three were not differentially expressed in soybean roots inoculated with rhizobia strains 113-2 and USDA205 (Table 4), and the detailed expression information of these nodulin genes at all the tested time points in the three groups was shown in Supplemental Table S4. However, their functions have not been well understood.

**Table 4:** **Twenty-five differentially expressed nodulin genes identified in soybean roots by RNA-Seq**.

**Gene name**	**Gene ID in soybean**	**Group 1**	**Group 2**	**Group 3**
Early nodulin-like protein 1	Glyma06g42110	+		
	Glyma12g32270	+		+
	Glyma12g34100	+		
	Glyma13g38150	+		
Early nodulin-like protein 2	Glyma12g13130	+		+
Nodulin-16	Glyma02g43320	+	+	+
	Glyma02g43330		+	+
Nodulin-20	Glyma13g40400	+	+	+
Nodulin-21	Glyma05g25010	+	+	+
	Glyma08g08120	+	+	
Nodulin-22	Glyma15g05010	+	+	+
Nodulin-24	Glyma02g43341	+	+	+
Nodulin-26	Glyma08g12650	+	+	+
	Glyma13g40820	+	+	+
Nodulin-36	Glyma01g03470	+	+	+
Nodulin-44	Glyma15g41445	+	+	
Nodulin-50	Glyma10g34280	+	+	+
Nodulin-51	Glyma20g02921	+	+	+
Nodulin-61	Glyma10g06810	+	+	+
Early nodulin-70	Glyma18g02230	+	+	+
Early nodulin-93	Glyma05g08400	+	+	+
	Glyma06g24760	+	+	
Other nodulins	Glyma02g04180	+	+	+
	Glyma06g06930	+	+	+
	Glyma17g08110	+	+	+


“*+” Indicates that the gene were different expressed in the group.*

### Verification of RNA-seq results by qPCR and expression analysis of 189 candidate genes in roots and/or nodules

**Figure 7:**
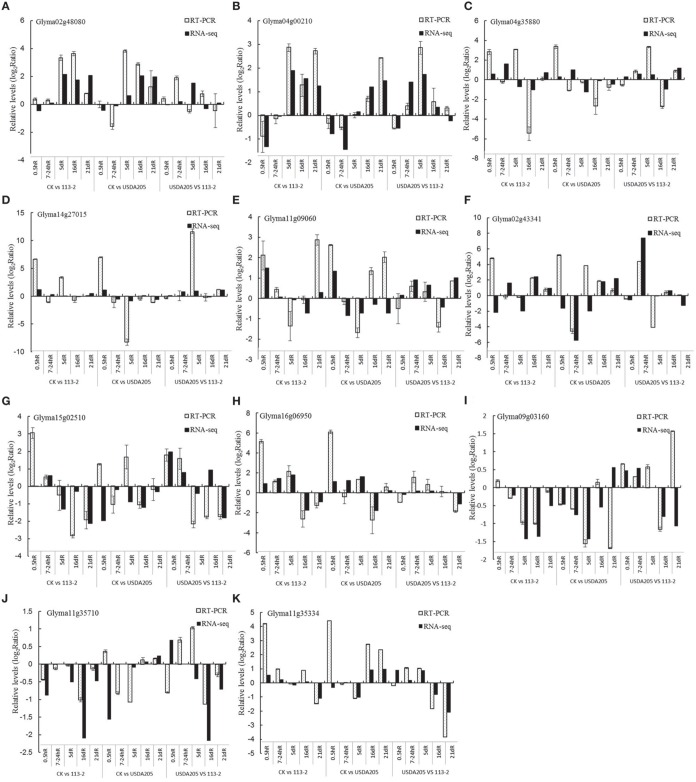
**Comparison of expression rates determined by RNA-Seq and qPCR on 11 genes in soybean roots**. All qPCR reactions were repeated three times and the data are presented as the mean ± SD. **(A)**, Glyma02g48080; **(B)**, Glyma04g00210; **(C)**, Glyma04g35880; **(D)**, Glyma14g27015; **(E)**, Glyma11g09060; **(F)**, Glyma02g43341; **(G)**, Glyma15g02510; **(H)**, Glyma16g06950; **(I)**, Glyma09g03160; **(J)**, Glyma11g35710; **(K)**, Glyma11g35334.

To verify the RNA-Seq results, the expression stability of five reference genes (ELF1b, Qact, G6PD, Fbox and Ubiquitin) was evaluated (Supplemental Figure S3), of which, Ubiquitin, ELF1b and Qact were most stable in all samples, while GmG6PD and Fbox were consistently unstable (Supplemental Figure S3). Thus, Ubiquitin was selected as reference gene for quantitative real-time PCR (qPCR) experiment. A total of 11 DEGs were randomly selected based on the transcriptional profile analysis and measured by qPCR. The results were in agreement with the transcriptional profile data for 136 out of 165 (82.42%) data points (Figure 7). Although, the fold-changes were not exactly identical, both methods yielded identical expression trends for most data points. The sequences of the specific primers used for qPCR are given in Supplemental Table S5.

To confirm the candidate genes whether or not play roles in nodulation, the expressions of 189 candidate genes (78 from Tables 1–3, others are protein kinases and/or transcription factors) in roots and/or nodules were analyzed according to two databases (Phytozome v10.3 and Soybase) (Supplemental Table S6). The results showed that only two genes (Glyma04g06470 and Glyma18g42610) nearly have no expression in roots and\ or nodules, indicating that most of these candidate genes might be indeed regulated or have a role in nodulation.

## Discussions

In this study, RNA-Seq was utilized to investigate the causes of different symbiotic phenotypes in soybean roots inoculated with different rhizobium strains *B. japonicum* 113-2 and *S. fredii* USDA205. RNA-Seq is an effective method that produces quantitative data related to transcripts with greater sensitivity, higher repeatability, and wider dynamic range (Jiang et al., 2014) than conventional methods. This method has also been shown to have relatively little variation between technical replicates to identify DEGs (Marioni et al., 2008). Consistent with the previous reports, our qPCR results agree with the transcriptional profile data for 136 out of 165(82.42%) data points (Figure 7), and 189 candidate genes are largely expressed in roots and\ or nodules (Supplemental Table S6), suggesting our RNA-Seq data are reliable.

The efficiency of symbiotic nitrogen fixation in soybean by application of inoculants greatly depends on the symbiotic host-specificity. To identify the genes that can control the host specificity and elucidate the molecular mechanisms for the host-restriction of nodulation, we focused on DEGs in response to different rhizobium strains (*B. japonicum* strain 113-2 and *S. fredii* strain USDA205) in soybean roots, and identified a large number of DEGs from RNA-Seq data. Our results for the first time identified DEGs that could be involved in the molecular events of nodulation in soybean roots inoculated with different strains. Among these DEGs, many are associated with the flavonoids biosynthesis pathway and the plant-pathogen interaction pathway.

### DEGs involved in nod factor and EPS signals transduction in soybean root cells

In the legume–rhizobium symbiosis, rhizobium ex-o-poly-saccharides (EPS), which act as nod factors, are also essential for bacterial infection (Jones et al., 2008). An EPS receptor (EPR3) identified in *L. japonicus* could selectively bind to compatible EPS to control rhizobium infection in bacterial competition studies (Kawaharada et al., 2015). The symbiotic specificity is thus regulated by a two-stage mechanism involving sequential receptor-mediated recognition and transduction of nod factors and EPS signals.

In connection with the soybean genome-based information and the information obtained from the model legumes, the nodulation signaling transduction pathway of soybean is clear and the NF-related genes are shown in Supplemental Table S7. In this report, the NF-related genes were not differentially expressed after 113-2 and/or USDA205 inoculation, except six in Group 1 and five in Group 2 (Table 3), suggesting that these genes were not changed at the five time points. Interestingly, *GmNIN-Like* genes were differentially expressed in the three groups. The results indicate that this gene was induced by nod factors and responded differently to different nod factors, which partly explains the differences in nodulation and/or nitrogen-fixation time and nodule numbers per root system between soybean roots inoculated with 113-2 and with USDA205 (Figure 1, Supplemental Table S1).

EPS signals are shared by pathogenic and symbiotic bacteria and play important roles in the legume–rhizobium symbiosis. However, their signal recognition and transduction mechanisms in soybean are not well explored (D'haeze and Holsters, 2004; Staehelin et al., 2006; Jones et al., 2008). In this report, we analyzed DEGs associated with flavonoids/flavone/flavonol biosynthesis pathway (Table 1) and plant-pathogen interaction pathway (Figure 5), with the hope that these DEGs might provide fundamental clues to the mechanisms underlying EPS signal recognition and transduction. The physiological activities of rhizobia are usually related to their EPS components, which change among different *E. fredii/S. fredii* and *B. japonicum* strains (Hotter and Scott, 1991). Thus, different EPS receptors may exist in legume for responses to different rhizobium strains.

### DEGs involved in the flavonoids/flavone/flavonol biosynthesis and plant immunity defense

Isoflavonoids, a subclass of much more common flavonoids, are the signals released by the soybean to attract rhizobium (Rolfe, 1988). They are secreted in host-specific manner, but formed by the same flavonoids biosynthetic pathway (Deavours and Dixon, 2005; Barnes, 2010), which is associated with flavone and flavonol biosynthesis pathway. The analyses of DEGs associated with the flavonoids/flavone/flavonol biosynthesis pathway indicated that the biosynthesis and secretion of isoflavonoids may be related to the recognition of different Rhizobia to host legume.

Rhizobia can adopt pathogenic systems to modulate the host range in a genotype-specific manner (Okazaki et al., 2013). For example, T3SS, which is known as an introducer of virulence factors from plant pathogens (Hueck, 1998), can be induced by legume-derived flavonoids, affecting symbiosis with host legumes (Okazaki et al., 2013). In this report, we analyzed the DEGs involved in the plant–pathogen interaction KEGG pathway in Group 3 (Figure 5, Table 2) and found that Ca^2+^ signal, MAPK cascade, hypersensitive response (HR) and defense related gene induction are associated with the molecular events of nodulation in soybean roots. These data uncovered some important genes in the tightly regulated soybean nodulation process that coordinates nod factors signal transduction with the host soybean immunity defense. However, the mechanism of this co-regulation remains to be determined.

### R genes differential expression in soybean roots inoculated with 113-2 or USDA205

Rhizobia can act as plant pathogens to infect legumes and cause a series of host immune responses (Okazaki et al., 2013), along with changes in expression of resistance (R) proteins inside soybean roots. Plant resistance (R) genes can specifically recognize the corresponding pathogen effectors or their associated protein(s) to activate plant immune responses at the site of infection (Liu et al., 2007), including a series of defense signaling cascades and pathogenesis-related (PR) gene expression (Durrant and Dong, 2004). In this report, 49 DEGs encoding broad resistance (R) proteins and six R-response genes were identified in soybean roots (Figure 6, Supplemental Table S3). The relationships among these six R-response genes and 49 R genes will help us to understand the immunity defenses mechanism in soybean roots. Their interactions will be determined by *in vitro* and *in vivo* assays.

In summary, to explain the different nodulation phenotypes, we analyzed the differential gene expression responses in uninoculated soybean roots and in soybean roots inoculated with 113-2 or USDA205 using RNA-seq, and found that DEGs associated with the flavonoids biosynthesis pathway and plant-pathogen interaction pathway could be used to understand the receptor-mediated recognition and transduction of nod factor and EPS signals. The DEGs uncovered in this study and their analyses shed new light on the host legume control of nodulation specificity, and provided a molecular basis for further investigations of the mechanisms underlying the host-specific manners of nod factor and EPS signals reception and transduction.

## Author contributions

SY, XZ designed this work, SY wrote the manuscript, SY, RL performed most of the experiments, SC, HC, CZ, LC, ZS, XZ, ZY, and DQ contributed substantially to the completion of this work. All the authors read and approved the final Manuscript.

### Conflict of interest statement

The authors declare that the research was conducted in the absence of any commercial or financial relationships that could be construed as a potential conflict of interest.

## Abbreviations

RNS: (The root nodule symbiosis)
T3SS: (the type III secretion system)
qPCR: (Quantitative real-time PCR)
DEGs: (Differentially Expressed Genes)
FDR: (False Discovery Rate)
HR: (Hypersensitive response)
WEGO: (Gene Ontology functional classification)
RH: (relative humidity)
CT: (cycle threshold).
