# Supplementary material for: RNA-Seq Analysis of Differential Gene Expression Responding to Different Rhizobium Strains in Soybean (Glycine max) Roots
Source: Front Plant Sci. 2016 May 30;7:721. doi: 10.3389/fpls.2016.00721 (PMC4885319; doi:10.3389/fpls.2016.00721)
Supplement: Supplementary file 1 [file Table1.DOCX]

**Supplemental Table S1 Analysis of symbiotic phenotypic in *B.japonicum* 113-2 – soybean and *S.fredii* USDA205 – soybean symbiosis.**

|  |  | | **mean value ± SE** |  |
| --- | --- | --- | --- | --- |
|  | **Control** | | **113-2** | **USDA205** |
| Chlorophyll content-12d SPAD(arbitrary units) | 29.336±0.587(n=30) | | 25.907±0.961(n=29) | 26.220±0.796(n=30) |
| Chlorophyll content-30d SPAD(arbitrary units) | 11.687±1.492(n=30) | | 23.076±1.207(n=29) | 12.547±1.099(n=30) |
| Chlorophyll content-42d SPAD(arbitrary units) |  | | 27.693±0.718(n=27) | 7.023±1.583(n=30) |
| Number of nodules per plant-12d | |  | 13.042±1.548(n=24) | 0.083±0.083(n=24) |
| Number of nodules per plant-42d | |  | 51.52±5.337(n=25) | 16.640±3.424(n=25) |
| Dry Weight of per nodule-42d(mg) | |  | 2.133±0.146(n=1288) | 7.037±0.927(n=472) |
