# Supplementary material for: RNA-Seq Analysis of Differential Gene Expression Responding to Different Rhizobium Strains in Soybean (Glycine max) Roots
Source: Front Plant Sci. 2016 May 30;7:721. doi: 10.3389/fpls.2016.00721 (PMC4885319; doi:10.3389/fpls.2016.00721)
Supplement: Supplementary file 5 [file Table5.DOCX]

**Supplemental Table S5 Primers for genes selected for RT-PCR analysis.**

| **Gene** | **Forward primer** | **Reverse primer** |  | **PCR efficiency** |
| --- | --- | --- | --- | --- |
| *Ubiquitin* | 5'-GCTCGTTGTGTAATGTTGG-3' | 5'-CGTAGGTGGGATATTAGAGT-3' | | 1.00113885 |
| *Glyma02g48080* | 5'- CGTTTCCATCCTAGGCGTC -3' | 5'-TGTAGGTAAGGCAAAGTTAG-3' | | 0.96350623 |
| *Glyma04g00210* | 5'-CCTTGTTAGAGTAATTAGCGC-3' | 5'-GCGAACAAATGCGATGGAG-3' | | 0.965873262 |
| *Glyma04g35880* | 5'- CTCAAAATTGATGCAGAAGCC-3' | 5'-CACACTTCTAACAGCAGTTAC-3' | | 0.951499485 |
| *Glyma14g27015* | 5'-TGGTAGAACAACGCTTGGC-3' | 5'-CAGTTAACTAATGTCACCG-3' | | 1.054666545 |
| *Glyma11g09060* | 5'-ATGTGCAATTTCCAATAGCC -3' | 5'GATGATGATACAAGTAGGAG--3' | | 1.014899584 |
| *Glyma02g43341* | 5'-GACGGTGTAGTCCATCAAT-3' | 5'-GTGGTAAGAAAGACACCGTC-3' | | 1.018539304 |
| *Glyma15g02510* | 5'- ACGCTTGCCCGAGATAAGAT-3' | 5'-CAATCACCAAGCCTAAGCAC-3' | | 0.960428888 |
| *Glyma16g06950* | 5'-ACATGATCCAGTTCTACCTG-3' | 5'-GTCATTTACTAACTTCACCC-3' | | 0.967171162 |
| *Glyma09g03160* | 5'-CAATGTTTCCCTCTGTTATCG-3' | 5'-GACTAAAGTTTCTAACTCCACG-3' | | 0.956213951 |
| *Glyma11g35334* | 5'-AGAGGATACAGACTCAAGTG-3' | 5'-GGTTACAAGGGCTGGAAAC-3' | | 1.005403283 |
| *Glyma11g35710* | 5'-TTGTATCCGAACAGGCTAC-3' | 5'-ATGTGCCTTGGAGGAAACAG-3' | | 1.054609211 |
| *Qact* | 5'-ATCTTGACTGAGCGTGGTTATTCC-3' | 5'-GCTGGTCCTGGCTGTCTCC-3' | | 0.891225378 |
| *ELF1b* | 5'-GTTGAAAAGCCAGGGGACA-3' | 5'-TCTTACCCCTTGAGCGTGG-3' | | 0.867157346 |
| *G6PD* | 5'-ACTCCTTGATACCGTTGTCCAT-3' | 5'-GTTTGTTATCCGCCTACAGCCT-3' | | 0.944700223 |
| *Fbox* | 5'-AGATAGGGAAATTGTGCAGGT-3' | 5'-CTAATGGCAATTGCAGCTCTC-3' | | 0.890122025 |
