# Supplementary material for: RNA-Seq Analysis of Differential Gene Expression Responding to Different Rhizobium Strains in Soybean (Glycine max) Roots
Source: Front Plant Sci. 2016 May 30;7:721. doi: 10.3389/fpls.2016.00721 (PMC4885319; doi:10.3389/fpls.2016.00721)
Supplement: Supplementary file 7 [file Table7.DOCX]

**Table S7: List of NF-related genes in soybean by searching for homologs of *M. truncatula* and/or *L. japonicus***

| *Gene name* | *M. truncatula* | *L. japonicus* | Gene ID in soybean |
| --- | --- | --- | --- |
| *LjNFR1/MtLYK3* | Medtr5g093450 | Lj2.CM0545.250.r2.m | Glyma14g05060 |
|  | Medtr5g093440 | Lj6.CM0041.460.r2.a | Glyma02g43860 |
|  | Medtr5g093730 |  | Glyma02g43850 |
|  | Medtr5g093410 |  |  |
| *LjNFR5/MtNFP* | Medtr5g018990 | Lj2.CM0323.400.r2.d | Glyma11g06740 |
|  | Medtr8g093910 |  | Glyma01g38560 |
| *LjSymRK/MtDMI2* | Medtr5g032400 | Lj2.CM0177.340.r2.m | Glyma09g33510 |
|  |  |  | Glyma01g02460 |
| *MtDMI1* | Medtr2g005620 | Lj6.CM0508.260.r2.m | Glyma12g28860 |
|  |  |  | Glyma16g00500 |
|  |  |  | Glyma19g45310 |
| *LjCCaMK/MtDMI3* | Medtr8g047760 | Lj3.LjT02O17.60.r2.m | Glyma15g35070 |
|  | Medtr5g009940 |  | Glyma08g24360 |
|  |  |  | Glyma10g11020 |
| *LjNUP133* |  | Lj2.CM0191.150.nc | Glyma14g01130 |
|  |  |  | Glyma02g47560 |
| *LjNUP85* |  | Lj1.CM0171.120.nc | Glyma17g27490 |
| *LjNENA* |  | Lj2.3v2088070.1 | Glyma18g10340 |
|  |  |  | Glyma08g43390 |
|  |  |  | Glyma08g43250 |
|  |  |  | Glyma18g10170 |
| *MtERN1* | Medtr7g102550 | Lj1.CM0104.2670.r2.m | Glyma16g04410 |
|  | Medtr6g031080 |  | Glyma19g29000 |
| *MtERN3* | Medtr6g015110 | Lj4.CM0046.750.r2.a | Glyma08g12130 |
|  | Medtr4g134350 |  | Glyma05g29011 |
| *LjNSP1/MtNSP1* | Medtr8g025000 | Lj3.CM0416.1260.r2.d | Glyma07g04430 |
|  | Medtr5g015580 |  | Glyma16g01020 |
|  | Medtr8g101580 |  | Glyma05g22460 |
| *LjNSP2/MtNSP2* | Medtr3g097800 | Lj1.CM1976.90.r2.m | Glyma04g43090 |
|  | Medtr5g065380 |  | Glyma06g11610 |
|  |  |  | Glyma13g02840 |
| *LjNIN/MtNIN* | Medtr5g106690 | Lj2.CM0102.250.r2.m | Glyma06g00240 |
|  |  |  | Glyma04g00210 |
|  |  |  | Glyma02g48080 |
| *LjCyclops/MtIPD3* | Medtr5g027010 | Lj2.CM0803.150.r2.m | Glyma01g35255 |
|  |  |  | Glyma09g34695 |
| *MtPUB1* | Medtr5g090510 |  | Glyma02g43190 |
| *LjRop6* |  | Lj0.3v0167719.1 | Glyma04g02530 |
|  |  |  | Glyma01g36880 |
|  |  |  | Glyma16g23340 |
| *MtSYMREM* | Medtr4g144240 |  | Glyma08g015190 |
|  |  |  | Glyma11g02740 |
| *MtSINA4* | Medtr3g091510 |  | Glyma06g13440 |
|  |  |  | Glyma04g41410 |
| *MtHMGR1* | Medtr5g026630 |  | Glyma11g09330 |
|  |  |  | Glyma02g44070 |
|  |  |  | Glyma09g32850 |
|  |  |  | Glyma14g05020 |
| *LjSIP1* |  | Lj1.CM0113.1720.r2.m | Glyma06g01640 |
|  |  |  | Glyma04g01560 |
| *LjSIP2* |  | Lj3.3v2040150.1 | Glyma08g23900 |
|  |  |  | Glyma07g00520 |
| *LjSIE3* |  | Lj0.3v0301709.1 | Glyma01g23830 |
|  |  |  | Glyma08g37050 |
| *Mt NADPH oxidase* | Medtr1g099800 |  | Glyma06g01640 |

Gene names of *M. truncatula* and/or *L. japonicus* as per the open research articles in which these genes were cloned and published, the articles were not shown; Gene name of *L. japonicus* were changed from chr to Lj for convenience, and "0" indicates no match to the genome. The Soybean Genome Database [http://soybase.org/], Phytozome Database [http://www.phytozome.net/soybean], the lotus Database [http://www.kazusa.or.jp/lotus/], plant GDB Database [http://www.plantgdb.org/] and NCBI-BLAST [http://blast.ncbi.nlm.nih.gov/] online resources were searched to identify the homologies.
