# Supplementary material for: RNA-Seq Analysis of Differential Gene Expression Responding to Different Rhizobium Strains in Soybean (Glycine max) Roots
Source: Front Plant Sci. 2016 May 30;7:721. doi: 10.3389/fpls.2016.00721 (PMC4885319; doi:10.3389/fpls.2016.00721)
Supplement: Supplementary file 9 [file Image2.PDF]

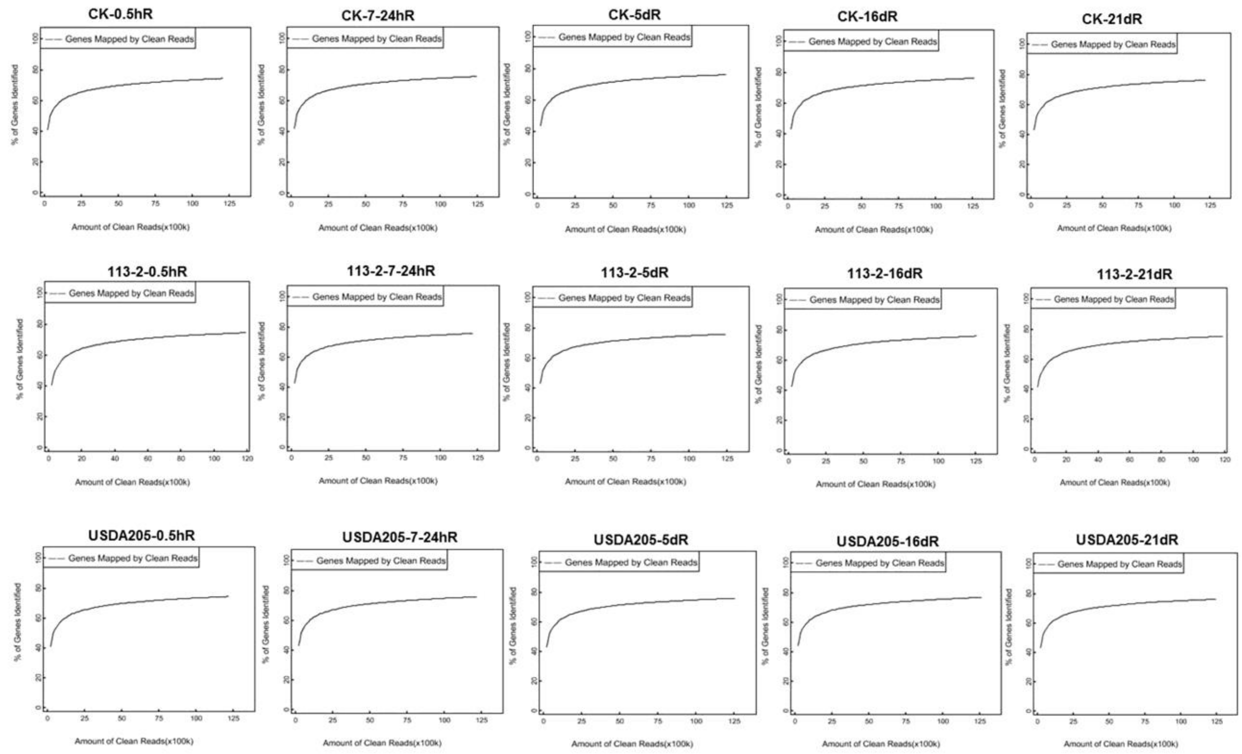

Supplemental Figure S2 Representative results of sequencing saturation analysis for soybean roots samples at five time points after inoculation with rhizobium strains 113-2 or USDA205.
