# Supplementary figures and images for: RNA-Seq Analysis of Differential Gene Expression Responding to Different Rhizobium Strains in Soybean (Glycine max) Roots
Source: Front Plant Sci. 2016 May 30;7:721. doi: 10.3389/fpls.2016.00721 (PMC4885319; doi:10.3389/fpls.2016.00721)

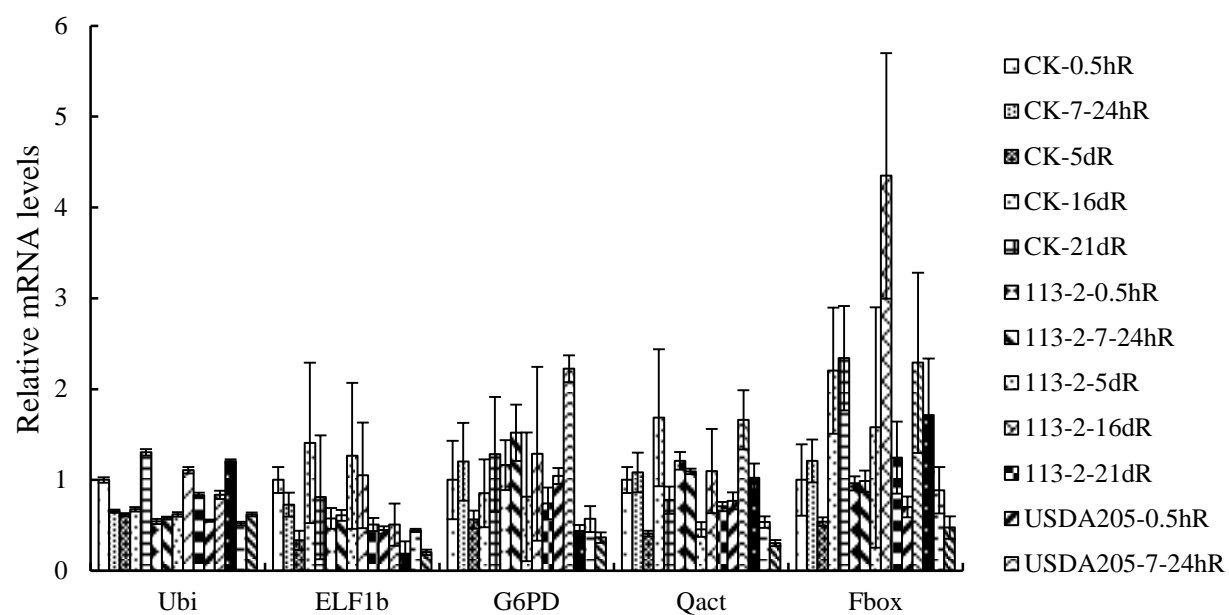

**Supplemental Figure S3 The stability assay of five references genes in 15 soybean roots samples.**

Supplement: Supplementary file 10 [file Image3.PDF]
